# Ranking Cancer Proteins by Integrating PPI Network and Protein Expression Profiles

**DOI:** 10.1155/2019/3907195

**Published:** 2019-01-06

**Authors:** Jie Ren, Lulu Shang, Qing Wang, Jing Li

**Affiliations:** ^1^Department of Bioinformatics and Biostatistics, School of Life Sciences and Biotechnology, Shanghai Jiao Tong University, Shanghai 200240, China; ^2^Department of Biostatistics, University of Michigan, 1415 Washington Heights, Ann Arbor, Michigan 48109-2029, USA

## Abstract

Proteomics, the large-scale analysis of proteins, is contributing greatly to understanding gene function in the postgenomic era. However, disease protein ranking using shotgun proteomics data has not been fully evaluated. In this study, we prioritized disease-related proteins by integrating the protein-protein interaction (PPI) network and protein differential expression profiles from colon and rectal cancer (CRC) or breast cancer (BC) proteomics. We applied Local Ranking (LR) and Global Ranking (GR) methods in network with three kinds of protein sets as a priori knowledge, which were known disease proteins (KDPs) that were collected from the Online Mendelian Inheritance in Man (OMIM) database, differentially expressed proteins (DEPs), and the collection of KDPs and their direct neighborhood with differential expression (eKDPs). The cross-validations showed that GR method outperformed LR method while using eKDPs as the initial training showed significantly higher accuracy compared to using the other two a priori sets. And then we validated the top ranked proteins using RNAi-based loss-of-function screens in the DepMap database. The results showed that 75% of top 20 proteins in CRC are necessary for tumor survival. In summary, the network-based Global Ranking with protein differential expression can efficiently prioritize cancer-related proteins and discover new candidate cancer genes or proteins.

## 1. Introduction

Discovering disease genes is important for understanding the mechanisms in physiological and pathological processes of disease. Genetic studies like linkage analysis [1] and association studies [2] can uncover diseases associated with chromosomal regions, which contain hundreds of candidate genes that may be associated with a disease [3]. Conducting experiments to confirm certain disease-related genes is time-consuming. To maximize efficiency, many computational methods and tools have been proposed to prioritize disease genes.

Since proteins are the basic unit of biological function to link genotypes to phenotypes, CPTAC and other cancer proteomics projects have characterized the proteomic features of human cancers [4–8]. However, how to prioritize cancer protein and discover new candidates based on PPI network and protein expression has not been fully addressed.

In the previous reports, the gene prioritization approach mainly depends on the similarity of characteristics, including sequence similarity [9, 10], function annotation [10–13], gene product [14], and protein domains, between known genes associated with the phenotype of interest and the candidate genes. In biological network, the neighbor genes may be functionally or physically similar and affect the pathway or the phenotype of interest [15]. Oti et al. [16] proposed a direct neighbor-based method to predict disease candidates with known disease loci. This method is based on the principle of “guilt-by-association” [17] and is called Local Ranking (LR) in this work. In addition to the LR method, Köhler et al. [18] proposed random walk method to access the global distance between the candidate genes and the known disease genes, which we called Global Ranking (GR) here. However, lack of knowledge about disease proteins and few known disease-related genes limits the development of the GR. To solve this problem, one approach combines the PPI network and the gene expression data to prioritize genes [19] like PINTA [20]. Le and Kwon [21] developed a neighbor-favoring weight reinforcement to improve the performance in disease gene prioritization. Recently, protein network-based integration of multiomics data for prioritizing cancer genes has also been proposed [22, 23]. However, these strategies have not been tested in proteomics data analysis yet.

In this study, by integrating PPI network and differentially expressed protein profiles given by shotgun proteomics, we evaluated LR and GR methods with three different initial protein sets as a priori knowledge to prioritize disease proteins. The three initial protein sets contain known disease proteins (KDPs), differentially expressed proteins (DEPs), and the KDPs with their direct neighbor DEPs (eKDPs). The cross-validations and RNAi perturbation screens were used for performance evaluation of different strategies.

## 2. Materials and Methods

### 2.1. Proteomic Datasets

To test and compare the two ranking methods with three initial protein sets, two cancer proteome datasets were used. The proteomic data of CRC used in our work include 90 TCGA colon tumor samples and 30 normal colon epithelium samples, and the normalized proteomic expression profile is provided in Zhang's work [4]. The other proteomic dataset contains 77 tumor samples and 3 normal breast tissue samples, and its normalization is presented in Mertins et al. [5]

### 2.2. Protein-Protein Interaction Network

We constructed protein interaction networks based on the HINT database (http://hint.yulab.org/). HINT [24] (High-quality INTeractomes) is a database of high-quality protein-protein interactions in different organisms and contains two types of interactions: binary physical interactions and cocomplex associations. After removing duplicates and self-linked interactions, we obtained 132032 interactions between 13048 proteins with the average degree of 10.12.

### 2.3. Cancer Protein List

The 44 known CRC genes were collected from the OMIM [25] knowledgebase (http://www.ncbi.nlm.nih.gov/omim), 39 of which exist in the HINT, as shown in Table 1. Similarly, 28 of 33 known BC genes collected from OMIM exist in HINT, as shown in Table 2.

### 2.4. Differential Expression Measures

The statistical analysis of protein differential expression was Welch's* t*-test, and a Bonferroni correction (p < 0.05) was applied, leading to 2455 DEPs in the CRC and 1549 DEPs in BC. We selected the DEPs directly linked with the KDPs in the PPI network to enlarge the initial protein set and made up the third initial protein set eKDPs.

### 2.5. Performance Measurement

Similarly to the previous disease gene ranking work [19], we employed a leave-one-out cross-validation test to validate the different models. In each iteration, one KDP and the other 99 non-KDP proteins were randomly selected to set up the testing set. All the remaining KDPs were used for training and building the model. A generalization error estimate was obtained by repeating this procedure for each of the KDPs available. Here only the proteins in the HINT PPI network were considered. The performance measurement used was the receiver-operating characteristic (ROC) analysis [26]. “roc” function in R package “pROC” was used to realize the measurement. The ranking scores of these proteins in the testing set given by the GR or LR method were used as predictor.

Genome-scale perturbation screens can now be efficiently performed in many cell lines using RNAi to knock down the target genes. Tsherniak et al. systematically analyzed genome-scale loss-function screens performed in 501 cancer cell lines and the results were publicly available on the Cancer Dependency web [27]. In order to validate the top ranked genes/proteins that we newly identified in CRC samples, we investigated their gene-level differential dependency scores (DS) at the web (https://depmap.org/rnai/index) to measure the relationship to tumor survival.

### 2.6. Ranking Strategies

In the LR method, the protein rankings are solely based on the directly connected protein neighbors, which can be one of the three kinds of initial protein sets (KDPs, DEPs, and eKDPs). The GR method can also use the information of the three kinds of initial protein sets by processing a random network walk on the PPI network.

The LR method sets the number of proteins in the initial protein set as its only standard for evaluating the probability of a candidate protein being associated with a disease. The more proteins in the initial protein set are associated with the candidate proteins, the higher score the candidate protein will get. In the GR method, the random walk restarts at one of the three kinds of initial protein sets. We adopted the heat kernel rank [28] method, which has been applied in gene prioritization previously [29].

Given a graph G, processing a random walk on G, the transition probability matrix W is defined as W=D^−1^A. A is the adjacency matrix and D is the diagonal matrix. The Laplacian matrix of G is L=I-W. By establishing the diffusion rate *α* and setting a preference vector *p*_0_, the ranking score vector can be got as the following Equation:

(1) $$\begin{array}{c} p_{\alpha }=p_{0}e^{-\alpha L} \end{array}$$

The similarity matrix of proteins is shown in

(2) $$\begin{array}{c} S={\left(\;I+\frac{-\partial }{N}L\;\right)}^{N} \end{array}$$

Then, we can obtain the discrete approximation (see (3)) described in Yang's work [30]:

(3) $$\begin{array}{c} p_{\alpha }=p_{0}{\left(\;I+\frac{-\alpha }{N}L\;\right)}^{N} \end{array}$$

where parameter *α* is the diffusion rate and N is the number of iterations. As the two parameters are set, a random walk through the network is initiated.

We set *α*=0.5 and N=3 for the interactions that can make *p*_∂_ reach a steady state. We initialized the preference vector *p*_0_ with binary values and filled the initial protein set with 1 and all the other candidate proteins with 0. Finally, we can obtain the vector *p*_∂_, which contained scores for all of the candidate proteins. It should be noted that the KDP's neighbor DEPs were not initialized when this KDP was selected for the testing set in leave-one-out cross-validation.

## 3. Results

### 3.1. Comparing LR, GR Methods with Three Different Kinds of Initial Protein Set

**Figure 1:**
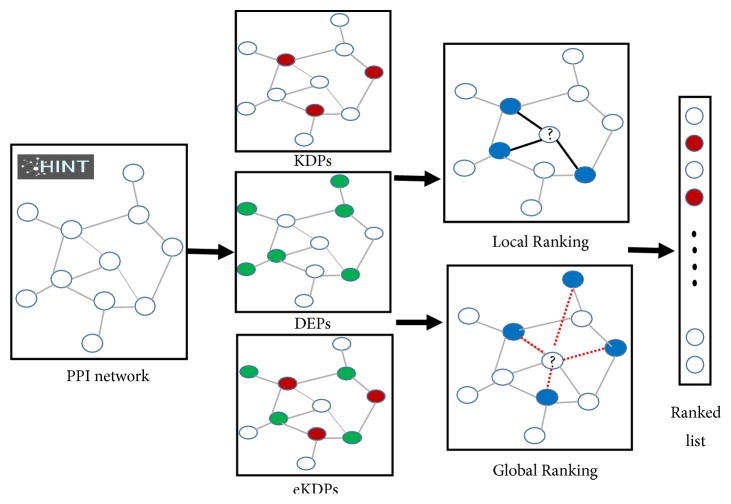
Workflow of the ranking process of LR and GR with three kinds of initial protein sets. Firstly, we mapped the three kinds of protein sets to the PPI network as initial protein datasets, which consisted of KDPs (red), DEPs (green), and eKDPs. Then, we applied the LR or GR to the network based on the initial protein set (blue).

We compared the two ranking methods using local [16] and global interaction information [29] or both. We plotted the ROC curves and computed the AUC values to evaluate LR and GR with three different initial protein sets, respectively [26, 31].  Figure 1 shows a workflow of the comparison of the two ranking strategies. The LR method scores the candidate protein by the number of directly linked initial proteins, and the GR method scores the candidate protein by all the initial proteins. As a result, all the candidate proteins on the PPI network can be scored.

**Figure 2:**
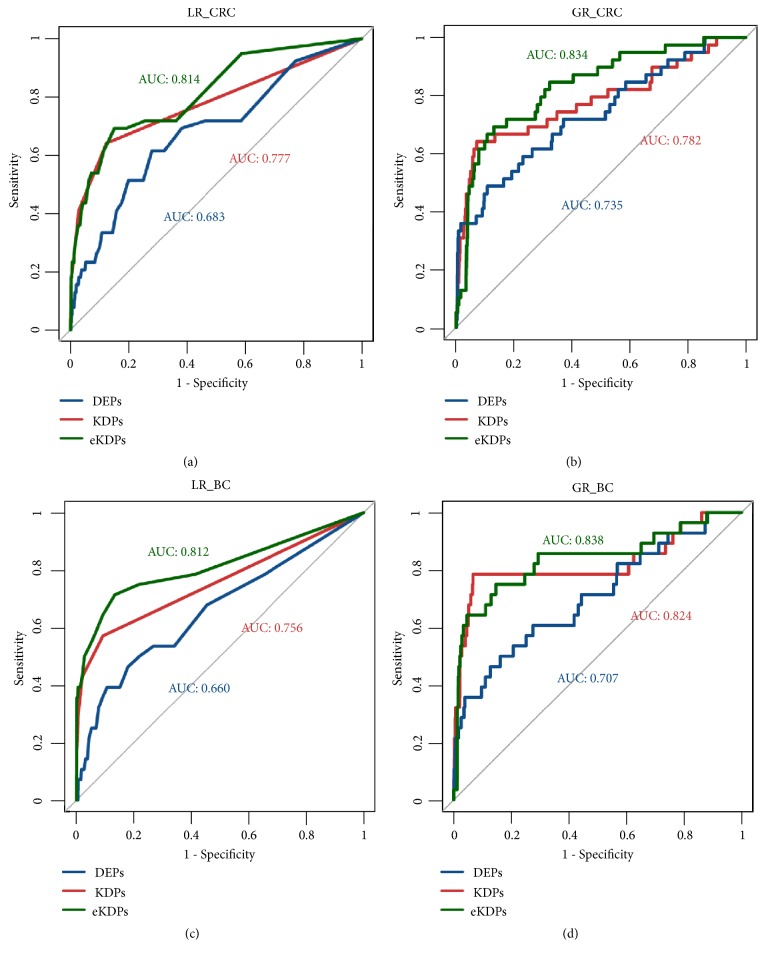
Comparison of LR and GR with three different initial protein sets. (a) and (b) show the results of CRC, and Figures (c) and (d) show the results of BC. KDPs (red), DEPs (blue), and DEPs and their neighbors (green) are the three initial protein sets.

In CRC, the AUC values of GR with different initial protein sets were 0.834, 0.782, and 0.735, better than the LR method, whose AUC values were 0.814, 0.777, and 0.683, respectively, as shown in Figure 2. As a result, using the same kind of prior knowledge, the performance of GR was better compared to LR. While using the eKDPs as the initial protein set, the result tended to be significantly better compared to using the other two datasets. We also calculated the AUC value of GR with the initial protein set involving the KDPs and 629 randomly selected DEPs (AUC=0.687). This result suggested that the GR method is suitable for proteome data as well as transcriptome data, and the neighbor DEPs can enlarge the initial protein set, which is helpful for ranking proteins.

The results of BC were the same as the results from CRC. These results showed that both the LR and GR methods can assess the similarity between two proteins in the PPI network for protein ranking. However, the GR method was better than the LR method.

**Figure 3:**
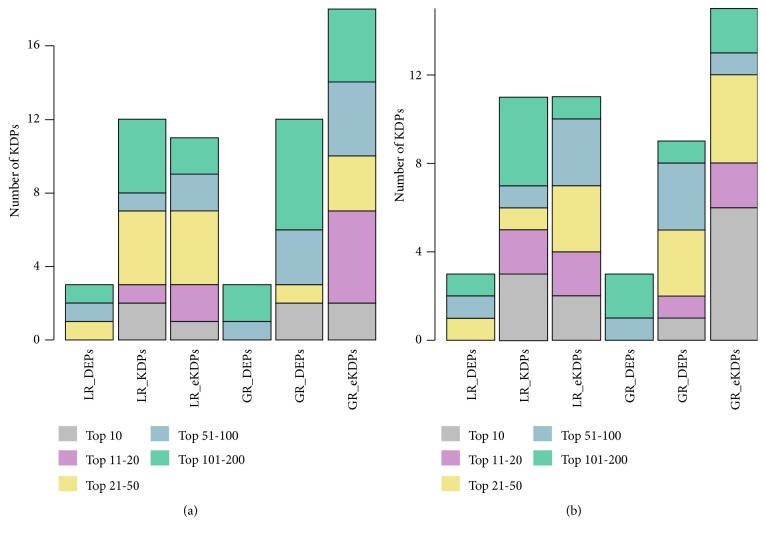
Comparison of the number of top ranked KDPs with different methods and initial protein sets. (a) shows the results of CRC and (b) shows the results of BC.

After prioritizing the proteins with the TCGA CRC shotgun proteomics, we obtained 6 ranked candidate protein lists for the LR and GR methods with three initial protein sets. We counted the number of KDPs ranked in the top 10, top 20, top 50, top 100, and top 200 proteins in the lists.  Figure 3 shows the comparison of the number of top ranked KDPs with different methods. We noted that the results of GR were the same as LR with the same initial protein set. However, while comparing the initial protein sets, eKDPs performed better than the other two. The top 100 ranked proteins resulted from GR method with eKDPs in CRC and BC are provided in Supplementary Table 1.

### 3.2. Annotation of the Top Ranked Proteins in CRC

**Figure 4:**
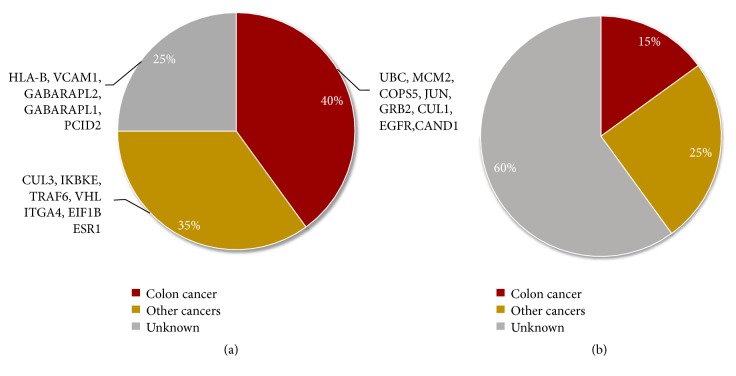
Cancer dependency evidences for the top 20 ranked proteins. The pie charts in (a) and (b) separately represent the percentage of top 20 ranked proteins excluding KDPs and 20 randomly selected candidate proteins, which are necessary for the survival of colon tumor or other cancers.. The gene annotation classes depend on the DS given by Cancer Dependency Map, which consists of colon cancer related proteins (DS<-2; red), other cancers related proteins (DS<-2; yellow), and proteins without relating to cancers or having no information (gray).

As the results shown above, the GR method with eKDPs performed better than the other two. We looked at the top 20 and top 50 ranked proteins/genes identified by the GR method with eKDPs in CRC samples. We found 7 KDPs in the top 20 ranked proteins (37%) and 10 KDPs in the top 50 ranked proteins (20%). Except for the KDPs, using the Cancer Dependency Map [27] we also queried the cancer dependency scores of 20 putative cancer genes/proteins in the top ranked list that we newly identified from the CRC dataset. As shown in Figure 4(a), 8 genes are necessary for colon tumor survival. In particular, UBC, MCM2, and COPS5 show stronger dependencies in colon cancer comparing with the mean across all cell lines (six sigma or greater dependency). Another seven genes have relationship with tumor survival in lung cancer, ovarian cancer, and other cancer types. In summary, 75% of the top 20 genes are necessary for the survival of colon cancer or other cancers' survival. At the same time, we selected 20 genes randomly from candidate protein list and repeated it 10 times. The average dependencies in colon and other types of cancers of these genes were illustrated in Figure 4(b). Only 40% in the 20 randomly selected candidate proteins are necessary for tumor survival, which is significantly lower than the proportion in our top ranked list (Pearson chi-square= 5.013, p= 0.025).

## 4. Discussion

In this manuscript, we compared the performance of network-based methods using protein expression profiles for cancer protein ranking. We found that the GR method outperformed when the eKDPs were used as the initial protein set. We randomly selected 39 proteins in the PPI network and compared with 39 KDPs for their degrees linking with the differentially expressed nodes. The average link of colon cancer proteins to DEPs was 16.46, which is significantly higher than the average value 5.65 from random controls. This result suggests that the expressions of the close neighbors of cancer proteins are more likely to be changed.

We further validated the top ranked proteins/genes in CRC samples based on the dependency score given by the Cancer Dependency Map. 75% of top 20 genes showed relationship with tumor survival. For example, UBC has different functions depending on the Lys residue linked by the ubiquitin. UBC is involved in DNA repair, kinase modification, protein degradation, DNA-damage responses, and other pathways [32]. One study showed Ubiquitin C-terminal hydrolase-L1 (UCHL1) can activate the *β*-catenin/TCF pathway through its deubiquitinating activity to contribute to CRC progression [33]. Minichromosome maintenance complex component 2 (MCM2) is involved in the initiation of eukaryotic genome replication. It has been reported that MCM2 is a therapeutic target of Trichostatin A in CRC cells [34] and also has been suggested to be used in the early diagnosis of CRC [35]. The Jun Protooncogene (JUN) is involved in activated KRAS-mediated transcriptional activation of USP28 in CRC cells, where it binds to the USP28 promoter [36]. Cullin 3 (CUL3) is the core component of multiple cullin-RING-based BCR (BTB-CUL3-RBX1) E3 ubiquitin-protein ligase complexes, which mediate the ubiquitination and subsequent proteasomal degradation of target proteins. As shown by Wang, CUL3 downregulation rescues folate deprivation-induced MAT II*α* exhaustion and growth arrest in CRC cells [37].

## 5. Conclusions

Global network-based ranking is more efficient for proteomics data in cancer protein identification. The network-based proteome analysis is helpful in ranking disease proteins and discovering new candidate proteins.

## Figures and Tables

**Table 1:** Known disease protein list of CRC also found in HINT.

Cancer type	Cancer genes (Entrez gene ID)
Colorectal Cancer	RAD54L; PTPN12; EP300; DLC1; CTNNB1; AURKA; MSH6; TGFBR2; BUB1B; SMAD7; CCND1; SRC; PTPRJ; PLA2G2A; POLD1; BRAF; BUB1; MLH3; MLH1; FLCN; BAX; MSH2; APC; RAD54B; GALNT12; CHEK2P1; AKT1; TP53; FGFR3; PIK3CA; PMS2; NRAS; cds1; AXIN2; MUTYH; MCC; TLR2; DCC; ODC1

**Table 2:** Known disease protein list of BC also found in HINT.

Cancer type	Cancer genes (Entrez gene ID)
Breast Cancer	XRCC3; RAD54L; casp8; BACH1; RAD51D; kras; ESR1; PALB2; NQO1; RAD51; RAD51C; TSG101; PPM1D; brca2; BARD1; BRCA1; PHB; AKT1; TP53; PIK3CA; RB1CC1; HMMR; NQO2; cds1; SLC22A18; ATM; BRIP1; CDH1

## Data Availability

The processed proteomic expression profiles supporting this study are from previously reported studies and datasets, which have been cited. The code used to support the findings of this study is available at https://github.com/Mathlida/protein-ranking.

## Abbreviations

LR: Local Ranking
GR: Global Ranking
CRC: Colon and rectal cancer
BC: Breast cancer
KDPs: Known disease proteins
DEPs: Differentially expressed proteins
eKDPs: KDPs with their neighbor DEPs
RWR: Random Walk with Restart
DS: Dependency Scores
HINT: High-quality INTeractomes
ROC: Receiver-operating characteristic
AUC: Area under the curve
UCHL1: Ubiquitin C-terminal hydrolase-L1
MCM2: Minichromosome maintenance complex component 2
JUN: Jun Protooncogene
CUL3: Cullin 3.

## Supplementary Materials

Supplementary Materials Supplementary Table 1: the top 100 ranked proteins that resulted from GR method with eKDPs in CRC and BC. The table lists the top 100 ranked proteins that resulted from GR method with eKDPs in colon and rectal cancer and breast cancer.
