# Supplementary material for: Ranking Cancer Proteins by Integrating PPI Network and Protein Expression Profiles
Source: Biomed Res Int. 2019 Jan 6;2019:3907195. doi: 10.1155/2019/3907195 (PMC6339728; doi:10.1155/2019/3907195)
Supplement: Supplementary Materials — Supplementary Table 1: the top 100 ranked proteins that resulted from GR method with eKDPs in CRC and BC. The table lists the top 100 ranked proteins that resulted from GR method with eKDPs in colon and rectal cancer and breast cancer. [file 3907195.f1.docx]

Supplementary Table 1: The top 100 ranked proteins resulted from GR method with eKDPs in CRC and BC

| Cancer type | Top 100 ranked proteins |
| --- | --- |
| Colorectal Cancer | JUN; MCC; APC; HLA-B; CUL3; UBC; MCM2; IKBKE; GRB2; TRAF6; TP53; AURKA; PIK3CA; COPS5; AKT1; GABARAPL2; EP300; GABARAPL1; VCAM1; VHL; CUL1; ITGA4; PCID2; EGFR; EIF1B; CTNNB1; CAND1; ESR1; SRC; NFKB1; PCNA; PRKAB1; MYC; NCK1; TMEM17; ZDHHC17; MAP1LC3A; MLH1; EIF6; STAT1; CRK; ABL1; HSP90AB1; MAP1LC3B; PIK3R1; YWHAB; TNIK; TOMM40; YWHAG; GSK3B; ARF6; MSH2; SMAD4; ATG5; FYN; XRCC6; GH1; YWHAZ; CUL2; ESR2; CDK2; EPB41; ILK; EEF1A1P9; CDKN1A; CRMP1; IKBKG; ERBB2; FTSJ1; MCM5; MAP3K3; CUL5; SCN2B; TGFBR2; PAK2; PSMB2; RAF1; IQCB1; TCTN3; CAPZA2; SUMO2P1; RAB7A; WWC1; TGFBR1; CDC42; CYP1A1; UBE2I; DCTN1; PINX1; RASA1; SHC1; RELA; RPGRIP1L; FBXW11; PTPN12; RPL10P15; MSH6; NOP56; COPS6; TMEM216 |
| Breast Cancer | AKT1; JUN; TP53; UBC; AARSD1; ATM; BRCA1; XRCC3; ESR1; RB1CC1; CUL3; HDAC1; YWHAZ; COPS5; MAP1LC3A; TSG101; PCID2; CAND1; C1orf94; YWHAB; MAP1LC3B; GABARAPL2; RAD51; LOC284685; YWHAG; EGFR; SCN2B; GABARAPL1; MCM2; GSK3B; AKT2; HLA-B; CA14; GRB2; VSIG2; SUMO2P1; BARD1; YWHAQ; TERF2IP; HSP90AB1; CASP8; PTPN1; HLA-DPA1; NRG1; MUC15; IKBKE; ITGA4; MMGT1; BACH1; CUL1; RSPH9; LIMK1; NCK1; VCAM1; RIF1; CDC37; SQSTM1; LYPD3; KAT5; PIK3R1; COPS6; EEF1A1P9; WDYHV1; SGTB; H2AFX; MCC; STAT6; YWHAH; AURKA; SETDB1; RB1; CHD3; MYC; ESR2; LGALS3BP; MAPK8; PIK3CA; MYO1C; AMOT; THOC2; SERPINB5; CDK2; SUMO1; SPDYA; THRSP; ILK; NAT2; SMAD4; USP11; HDAC2; BCL2L1; PLK1; UNC119; CMTM5; TRAF2; UNC13D; PPP1CA; PIK3R2; VIM; TMEM63B |
